# Ketone Administration for Seizure Disorders: History and Rationale for Ketone Esters and Metabolic Alternatives

**DOI:** 10.3389/fnins.2019.01041

**Published:** 2019-10-15

**Authors:** Angela M. Poff, Jong M. Rho, Dominic P. D’Agostino

**Affiliations:** ^1^Laboratory of Metabolic Medicine, Department of Molecular Pharmacology and Physiology, Morsani College of Medicine, University of South Florida, Tampa, FL, United States; ^2^Departments of Pediatrics, Clinical Neurosciences, Physiology and Pharmacology, Alberta Children’s Hospital Research Institute, Hotchkiss Brain Institute, Cumming School of Medicine, University of Calgary, Calgary, AB, Canada; ^3^Division of Pediatric Neurology, Rady Children’s Hospital-San Diego, University of California, San Diego, San Diego, CA, United States; ^4^Institute for Human and Machine Cognition, Ocala, FL, United States

**Keywords:** ketogenic diet, metabolic therapy, beta-hydroxybutyrate, acetoacetate, ketosis, exogenous ketones, ketone esters, epilepsy

## Abstract

The ketogenic diet (KD) is a high-fat, low-carbohydrate treatment for medically intractable epilepsy. One of the hallmark features of the KD is the production of ketone bodies which have long been believed, but not yet proven, to exert direct anti-seizure effects. The prevailing view has been that ketosis is an epiphenomenon during KD treatment, mostly due to clinical observations that blood ketone levels do not correlate well with seizure control. Nevertheless, there is increasing experimental evidence that ketone bodies alone can exert anti-seizure properties through a multiplicity of mechanisms, including but not limited to: (1) activation of inhibitory adenosine and ATP-sensitive potassium channels; (2) enhancement of mitochondrial function and reduction in oxidative stress; (3) attenuation of excitatory neurotransmission; and (4) enhancement of central γ-aminobutyric acid (GABA) synthesis. Other novel actions more recently reported include inhibition of inflammasome assembly and activation of peripheral immune cells, and epigenetic effects by decreasing the activity of histone deacetylases (HDACs). Collectively, the preclinical evidence to date suggests that ketone administration alone might afford anti-seizure benefits for patients with epilepsy. There are, however, pragmatic challenges in administering ketone bodies in humans, but prior concerns may largely be mitigated through the use of ketone esters or balanced ketone electrolyte formulations that can be given orally and induce elevated and sustained hyperketonemia to achieve therapeutic effects.

## KEY POINTS

- Cellular metabolism plays a key role in the modulation of neuronal excitability.
- The high-fat, low-carbohydrate ketogenic diet (KD) is a validated treatment for persons with epilepsy. and is also effective in preventing seizures in animal models.
- Beta-hydroxybutyrate (βHB) and acetoacetate (AcAc), the ketone bodies that increase during KD treatment, exert anti-seizure effects in animal models of epilepsy and neurometabolic disorders.
- Although human clinical trials are still needed, therapeutic ketosis with ketone esters represents a clinically viable formulation for the potential treatment of epilepsy and other seizure disorders.

## Introduction

The traditional paradigm for discovery of new anti-seizure drugs (ASDs, also referred to as antiepileptic drugs or AEDs) has involved the assessment of agents blocking acutely provoked or kindled seizures, and which has led to the development of medications that largely influence cellular membrane-bound ion channels and transporters localized to synapses in the central nervous system (CNS) (Rogawski et al., 2016). More recently, however, it has become clear that metabolic factors – whether substrates or enzymes involved in cellular bioenergetics and metabolism – can profoundly influence neuronal excitability (Rogawski, 2016). Research linking brain metabolic changes to neuronal excitability has been driven by efforts to understand how the high-fat, low-carbohydrate ketogenic diet (KD) exerts its anti-seizure – and potential neuroprotective – effects in persons with epilepsy (Neal et al., 2008; Tanner et al., 2011; Rho and Stafstrom, 2012; Stafstrom and Rho, 2012; Gano et al., 2014; Rogawski et al., 2016).

While the efficacy of the KD in the clinical arena is clearly established (Freeman et al., 1998; Neal et al., 2008, 2009; Lambrechts et al., 2017), the mechanisms underlying its beneficial effects remain incompletely understood. Of the many hypotheses proposed (Rogawski et al., 2016), – a historical and unresolved question – is whether ketone bodies (i.e., β-hydroxybutyrate [βHB], acetoacetate [AcAc] and acetone [ACE]) are direct mediators or whether they are epiphenomena, instead indicative of a shift from glycolysis to fatty acid oxidation. Certainly, the current human clinical data do not yet strongly support the view that ketone bodies possess anti-seizure properties independent of their serving as fuel for ATP production, mostly because clinical and a few experimental studies have shown that blood ketone levels do not correlate directly with seizure control (Gilbert et al., 2000; Thavendiranathan et al., 2000; van Delft et al., 2010; Dallerac et al., 2017), despite increasing evidence in the preclinical literature (Kim et al., 2015; Simeone et al., 2018) and more recent clinical evidence to the contrary (Buchhalter et al., 2017). And all three major ketones (βHB, AcAc and ACE) have been shown to have anti-seizure effects in prior animal studies (Keith, 1931; Rho et al., 2002; Likhodii et al., 2003; Kim et al., 2015). For a comprehensive review of ketone bodies as anti-seizure agents, see Simeone et al. (2018). In this manuscript, we review the literature surrounding exogenous administration of ketogenic agents as a potential anti-seizure therapy. As the field is in its infancy, there is little published clinical data available; therefore, we place particular focus on the scientific rationale and pre-clinical evidence which support the translation of these therapies into currently ongoing and future human trials.

## Ketone Metabolism

The pathways involved with ketone body synthesis and metabolism have been firmly established for decades. Fatty acids are converted to acetyl-CoA which then enters the tricarboxylic acid (TCA) cycle. Under conditions where fatty acid levels increase and exceed maximal TCA cycle function, such as during fasting or treatment with the KD, acetyl-CoA is diverted to ketogenesis. Two molecules of acetyl-CoA are used to form acetoacetyl-CoA via acetoacetyl-CoA thiolase. Acetoacetyl-CoA is then condensed with another molecule of acetyl-CoA to form 3-hydroxy-3-methylglutaryl CoA (HMGCoA) in a non-reversible step catalyzed by the rate-limiting enzyme HMG-CoA synthase 2 (HMG-CoAS2). The ketone body AcAc is then produced via the breakdown of HMG-CoA, which releases a molecule of acetyl-CoA. AcAc in turn can either be interconverted to βHB through the βHB-dehydrogenase enzyme or can be spontaneously decarboxylated to acetone and released primarily through the kidneys or lungs. Ketone bodies can then pass through the blood-brain-barrier through monocarboxylic acid transporters (MCTs) and enter the brain interstitial space. After being transported into mitochondria, ketone bodies can be converted back through several enzymatic steps to acetyl-CoA which enters the TCA cycle in neurons or glia to produce ATP. Alternatively, ketone bodies may exert other biological effects such as those described below.

## Evidence for the Anti-Seizure Effects of Ketone Bodies

Not surprisingly, given the key hallmark feature of the KD is systemic ketosis, investigators focused on ketone bodies as possible mediators of anti-seizure effects. Indeed, ketone bodies were shown as early as the 1930’s to protect against acutely provoked seizures in rabbits (Keith, 1931, 1932, 1933, 1935), findings that were replicated and expanded decades later in multiple rodent models of seizures and epilepsy (Likhodii and Burnham, 2002; Rho et al., 2002; Likhodii et al., 2003; Minlebaev and Khazipov, 2011; Kim et al., 2015; Yum et al., 2015). Notably, *in vivo* anti-seizure effects were reported for either BHB, AcAc or ACE. However, the question of whether one or a combination of these ketone bodies affords even greater efficacy has not been answered. Taken together, these and other studies provide compelling evidence that ketone bodies can induce significant anti-seizure effects, and thus one cannot readily dismiss the possibility that these metabolic substrates contribute directly to the clinical effects of the KD.

In contrast to preclinical data referenced above, ketone bodies (when administered *in vitro* at low millimolar concentrations) were unable to affect either excitatory or inhibitory hippocampal synaptic transmission (Thio et al., 2000) and did not affect voltage-gated sodium channels (Yang et al., 2007) in normal hippocampus, unlike how current anti-seizure drugs are believed to generally work (Rogawski et al., 2016). Notwithstanding these observations, there are two aspects of ketone body action that overlap with synaptic function, but in distinct ways. First, ketone bodies (notably, AcAc) were shown to block neuronal excitability and seizures by inhibiting the presynaptic release of glutamate by modulating vesicular glutamate transporters or VGLUTs (Juge et al., 2010). Second, BHB was shown to alter the aspartate-to-glutamate ratio by driving the aspartate aminotransferase reaction (specifically, by decreasing the transamination of glutamate to aspartate) such that glutamate decarboxylation to GABA is increased (Erecinska et al., 1996; Daikhin and Yudkoff, 1998). The increase in GABA production would then be expected to enhance inhibitory neurotransmission and dampen seizure activity. Despite the rational neurochemical data, more direct evidence for this mechanism has not emerged (Yudkoff et al., 2001; Lund et al., 2009, 2011; Valente-Silva et al., 2015; Zhang et al., 2015), and this GABAergic hypothesis of ketone body action has not been reconciled with the fact that patients with epilepsy who were refractory to GABAergic medications often respond to the KD (Freeman et al., 2006).

The central challenge within the field of diet-based treatments for epilepsy has been to demonstrate clear causal links between cellular metabolism and plasmalemmal membrane excitability. A strong candidate molecular target was discussed nearly 20 years ago, i.e., ATP-sensitive potassium channels that, when activated by reduced ATP-to-ADP ratios, cause membrane hyperpolarization (Schwartzkroin, 1999). Using brain slices from normal and genetically engineered mice, Yellen and colleagues (Ma et al., 2007) showed that ketone bodies decreased the spontaneous firing of GABAergic interneurons in the substantia nigra pars reticulata (which is a known subcortical modulator of seizure propagation in the brain). Moreover, they demonstrated that this action required K_ATP_ channels and GABA_B_ receptors (Ma et al., 2007). However, it remains unclear whether K_ATP_ channels can be directly activated by ketone bodies, as other investigators have shown that both the KD and ketone bodies increase cellular levels of ATP, which would inhibit K_ATP_ channel opening (DeVivo et al., 1978; Bough et al., 2006; Kim et al., 2010). One potential mechanism reconciling these discrepant observations was provided by Kawamura et al. (2010) a few years later. These investigators showed that under low-glucose conditions (observed during KD treatment), ATP efflux from pyramidal neurons in CA3 hippocampus leads to conversion of ATP to adenosine by ectonucleotidase enzymes and subsequent activation of inhibitory adenosine receptors (A1Rs) which are coupled to K_ATP_ channel activation (Kawamura et al., 2010).

In more recent years, other novel targets for ketone body action have been reported. Kim et al. (2015) reported that BHB blocks spontaneous recurrent seizures in the *Kcna1*-null mouse model of epilepsy, and does so by inhibiting mitochondrial permeability transition – a critical death switch for the cell (Izzo et al., 2016). Further, while other studies have revealed ever increasing complexity of ketone body action on biological targets, they involved non-epileptic and/or extra-CNS tissues. Among the most intriguing are the following: (1) systemic anti-inflammatory effects induced by BHB via inhibition of nucleotide-binding domain (NOD)-like receptor protein 3 (NLRP3) inflammasome assembly (Youm et al., 2015); (2) neuroprotective and anti-inflammatory effects of BHB through an interaction with the hydroxycarboxylic acid 2 (HCA2) receptor (Rahman et al., 2014); and (3) inhibition of histone deacetylases (HDACs) and anti-oxidant effects in renal tissue by BHB (Shimazu et al., 2013). All of these mechanisms, although incompletely understood in the context of epileptic brain, expand the biological profile of BHB and provide further evidence that a strategy based on ketone body administration or inducing prominent ketosis might yield significant and measurable anti-seizure effects in the clinical setting.

## History and Pragmatic Challenges of Implementing Therapeutic Ketosis – Rationale for Ketone Esters

Administering the KD to implement therapeutic ketosis for seizure disorders is not without its challenges. The restrictive and precise macronutrient composition required to maintain and sustain nutritional ketosis can be difficult to implement. So while fundamental research may be spurred by the intrinsic curiosity and appeal of understanding how a dietary treatment can control epileptic seizures, a longstanding goal has been to determine whether a “KD in a pill” could be developed (Rho and Sankar, 2008). Indeed, investigators have sought ways to circumvent conventional means to administer the KD, one through more liberal and less restrictive diets such as the modified Atkins diet (Eric Kossoff et al., 2016) or the Low-Glycemic Index Therapy (LGIT) (Muzykewicz et al., 2009). Experimentally, other researchers have focused on ketone bodies and pragmatic formulations that could eventually be administered to humans to safely induce a dose-dependent and therapeutic hyperketonemia (Veech, 2004; D’Agostino et al., 2013; Hashim and VanItallie, 2014).

The idea of administering a ketogenic agent to induce and sustain therapeutic ketosis for parenteral and oral nutrition has been around for decades (Miller and Dymsza, 1967). Researchers in the 1950s at Massachusetts Institute of Technology, in collaboration with the Air Force Research Laboratory (AFRL), focused their efforts on high energy-dense compounds that had the greatest nutritional potential for long-duration manned spaceflight (Bornmann, 1954). Numerous agents were tested, but the ketogenic compound R,S-1,3-butanediol (BD; also known as R,S-1,3-butylene glycol) was selected as the most promising energy source, leading to further studies to determine its safety, stability, and potential as a food additive and preservative (Dymsza, 1975). Data were collected on rodents, dogs, pigs, and humans given this ketogenic compound, and although it induced hypoglycemia concomitant with ketonemia, it was deemed remarkably safe. R,S-1,3-butanediol met the criteria needed for the optimal synthetic “space food”, but the “unpleasant taste problem” and lack of FDA approval prevented its use for military or space flight applications.

Despite the palatability challenges, investigators remained intrigued with the potential applications of BD given its metabolic characteristics that mimicked fasting – mild hypoglycemia and safe and predictable hyperketonemia. When ingested orally, BD is metabolized by the liver via alcohol dehydrogenase (ADH) to β-hydroxybutyraldehyde, which is then rapidly oxidized to BHB by aldehyde dehydrogenase (Tate et al., 1971). BD contributes approximately 6 kcal/gm of energy and can produce dose-dependent millimolar concentrations of ketones in the blood at a ratio of 6:1 of BHB to AcAc (Tobin et al., 1972; Desrochers et al., 1992; D’Agostino et al., 2013). Published studies and a number of unpublished reports pertaining to the nutritional and metabolic effects of BD, including a human clinical study feeding study (young male and female subjects given 250 mg/kg body weight per day in bread for four separate 7-day periods), reported a blood glucose lowering effect as well (12% lower relative to controls) (Tobin et al., 1975). This was presumably due to a redox shift in the liver suppressing gluconeogenesis (Ciraolo and Previs, 1995). Although the mild hypoglycemic effect was a potential concern, extensive toxicology studies concluded that BD is safe with very few adverse health effects in animals and humans (Scala and Paynter, 1967; Dymsza, 1975; Hess et al., 1981). Consequently, it was given the status of being Generally Recognized As Safe (GRAS) in May 1997 by the FDA (Docket No. 87G-0351).

The early extensive safety and feasibility studies of BD, its FDA GRAS status, and high stability (i.e., shelf-life) inspired chemists and researchers to use BD as a backbone for synthesizing ketone esters (Brunengraber, 1997; Veech et al., 2001). Chemical synthesis by adding ketones (either BHB or ACAC) to this ketogenic diol through transesterification makes the resulting ketone esters the most energy-dense ketogenic supplements on a per gram basis. In addition to BD-derived ketone esters, there also exist glycerol-derived ketone esters of BHB. The diol BD and triol glycerol contain two or three hydroxyl groups, respectively, and through transesterification, these functional groups can pair with ketone bodies to make mono-esters, di-esters, or such as in the case of glycerol, a tri-ester compound known as glyceryl-tris-3-hydroxybutyrate (Hashim and VanItallie, 2014). Although deriving ketone esters utilizing glycerol as a backbone is feasible (Birkhahn and Border, 1978), the simultaneous elevation of glucose (glycerol is a gluconeogenic precursor) upon hydrolysis and subsequent increase in glycolysis can be unfavorable in the context of inducing anti-seizure effects. The advantage of BD as a backbone is that it delivers ketones upon esterase hydrolysis (in both gut and liver) and also metabolizes completely to BHB to further elevate and sustain ketosis in a predictable manner. Furthermore, dietary interventions that reduce glucose availability, Muzykewicz et al. (2009) and drugs targeting glycolysis such as 2-deoxyglucose (2-DG), Stafstrom et al. (2009) induce anti-seizure effects independent of ketone elevation, so mild hypoglycemia as a “side-effect” is theoretically advantageous for choosing ketogenic supplements that can be effective in controlling epileptic seizures.

The BD-derived ketone esters have been shown to induce a dose-dependent hyperketonemia (1–7 mM) in mice, rats, dogs, pigs, and humans (Desrochers et al., 1995; Clarke et al., 2012; Pascual et al., 2014; Newport et al., 2015). The emerging data indicate that these compounds produce no negative health effects when given acutely or chronically, aside from an aversive taste and the potential for dose-dependent gastrointestinal side effects. There are a growing number of promising metabolic alternatives to ketone esters that have improved or neutral taste and are considerably less expensive to produce. Emerging ketogenic supplements and formulas are being evaluated for their therapeutic efficacy (Borges and Sonnewald, 2012; Kesl et al., 2016) and their anti-seizure potential is discussed below (Table 1).

**TABLE 1:** Studies evaluating anticonvulsant efficacy of ketogenic agents in pre-clinical models.

**Authors**	**Ketogenic agent**	**Route of admin**	**Species**	**Model System**	**Result**	**References**
Rho et al., 2002	AcAc, ACE, L-BHB	i.p.	Mice	Frings audiogenic-induced Sz	↑Latency to Sz(D-BHIB no efect)	Rho et al., 2002
Likhodii et al., 2003	ACE	i.p. (acute) Oral in H20 (chronic)	Rat	PTZ-induced Sz	↑Sz threshold ↓Sz activity	Likhodii and Burnham, 2002
Likhodii et al., 2003	ACE	i.p.	Rat	Maximal Electroshock Test Amygdala Kindling Test AY-9944Test	↑Sz threshold ↓Sz activity	Likhodii et al., 2003
Minlebaev and Khazipov, 2011	DL-βHB	i.p.	Suckling Rat	Fluorthyl-induced Sz	↓Sz activity	Minlebaev and Khazipov, 2011
Wlaz et al., 2012	Caprylic Acid (C8)	Oral	Mice	i.v. PTZ-induced Sz 6Hz Psychomotor Sz Maximal Electroshock Test	↑Sz threshold (6Hz& i.v. PTZ Sz) No effect MEST	Wlaz et al., 2012
D’Agostino et al., 2013	BD-AcAc_2_	Oral	Rat	Hyperbaric Hyperoxia-induced CNS-OTSz	↑Latency to Sz	D’Agostino et al., 2013
Wlaz et al., 2015	Capric Acid (C10)	Oral	Mice	6Hz Psychomotor Sz Maximal Electroshock Test i.v. PTZ-induced Sz	↑Sz threshold (6Hz & MEST) No effect i.v. PTZSz	Wlaz et al., 2015
Viggiano et al., 2015	BD-ACAL_2_	Oral	Rat	PTZ-induced Sz	↑TPTZ threshold for Sz induction	Viggiano et al., 2015
Yum et al., 2015	D-βHB	i.p. (acute and chronic)	Rat	NMDA-induced Sz	No effect (acute) ↓Sz frequency (chronic)	Yum et al., 2015
Kim et al., 2015	BHB	s.c. (osmotic pump)	Mice	Kcna-null Mutant Mice	↓Sz frequency (*in vivo*) ↓Spontaneous Sz-like events (hippocampal slice)	Kim et al., 2015
Chang et al., 2015	4-ethyloctanoid acid (4-EOA)	Oral	Mice	6Hz Psychomotor Sz Maximal Electroshock Test S.c. Metrazol Sz Threshold Test Corneal Kindled Mouse Model	↑Sz control ↑Sz threshold	Chang et al., 2015
Kovacs et al., 2017	BD-AcAc_2_ KS-MCT	Oral	Rat	WAG/Rjj rats, absent Sz	↓Spike Wave Discharges	Kovacs et al., 2017
Ciarlone et al., 2016	BD-AcAc_z_	Oral	Mice	Ube3a m-/p+ and WT mice Audiogenic-induced Sz Kanic acid-induced Sz	↑Latency to Sz ↓Sz Activity ↓Sz Severity	Ciarlone et al., 2016

*A number of pre-clinical studies in varied seizure models have revealed anticonvulsant effects of exogenous ketogenic agents, including ketone salts, MCTs, and ketone esters.*

## Evidence for the Efficacy of Ketogenic Agent-Based Therapies for Epilepsy

**FIGURE 1:**
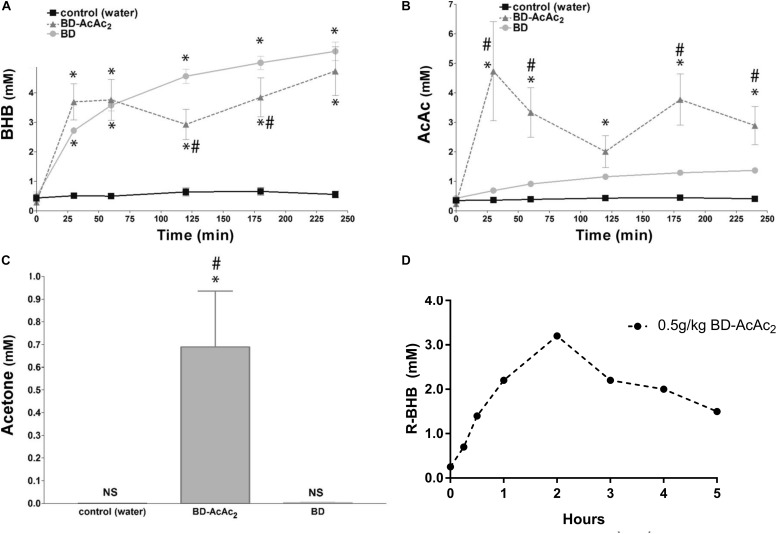
Exogenous ketogenic agents induce therapeutic ketosis within hours of oral ingestion. **(A–C)** Blood ketones following intragastric administration (time 0) of water, R,S-1,3-butanediol acetoacetate diester (BD-AcAc2), and 1,3-butanediol (BD) in Sprague Dawley rats. **(A)** β-hydroxybutyrate (βHB) level was elevated compared with control after either ketogenic compound (*P* < 0.001). **(B**) Acetoacetate (AcAc) level was increased significantly by BD-AcAc2 (*P* < 0.001) compared with water or BD. **(C)** Acetone level increased significantly more after treatment with BD-AcAc2 (*P* < 0.001). **(D)** Blood ketones (R-BHB only) following intragastric administration (time 0) of BD-AcAc2 in male human (*n* = 1). Figure adapted with permission from D’Agostino et al. (2013), American Physiological Society. ^∗^Significant difference from control and ^#^significant difference from BD.

The science and clinical applications of therapeutic ketosis for neurological applications is growing rapidly, but work evaluating exogenous ketogenic agents remains largely in the pre-clinical space (Stafstrom and Rho, 2012). In addition to ketone esters, there are numerous alternative sources of ketones and ketogenic precursors being developed and shown to produce dose-dependent elevations in blood BHB and AcAc in animals, human case report and pilot studies (Puchowicz et al., 2000; Clarke et al., 2012; D’Agostino et al., 2013; Kesl et al., 2016). Ketone supplemental therapies allow for a calculated, rapid induction and maintenance of physiologic ketosis that mimics levels associated with KD treatment for epileptic seizures (Figure 1). In humans it is likely that 2–3 doses/day would be needed to maintain therapeutic hyperketonemia. Ketone supplementation also appears to fundamentally shift metabolic physiology and fuel utilization (Cox et al., 2016), so its potential for supporting physical endurance and military applications is emerging. Since metabolic shifts can affect so many cellular and molecular processes simultaneously, it is not surprising that there is a growing list of mechanisms that have been implicated for exogenous ketones, as previously discussed. However, efficacy may vary depending on the model and endpoints utilized, as well as the physicochemical and pharmacological properties of the individual ketogenic agents and formulations.

## Ketone Esters

Currently, it appears that certain ketone esters hold great anti-seizure potential based upon previous work and on their pharmacokinetic profiles, although the current limitation is that research to date has largely been performed in pre-clinical animal models. When ketone esters are administered, gastric esterases liberate ketones (BHB or AcAc) as a free acid from a backbone molecule. This varies depending upon the specific formulation, but a ketogenic precursor such as BD would be ideal. As previously discussed, BD is subsequently metabolized by the liver to produce BHB (D’Agostino et al., 2013). Thus, the ketone esters currently available are unique in that they can directly elevate ketones and supply ketogenic precursors that can favorably change metabolic parameters like the glucose-ketone index (GKI) (Meidenbauer et al., 2015). Additionally, synthetically derived ketone esters are currently the most potent form of exogenous ketones available, but their potency also necessitates a thorough investigation of their long-term safety and toxicity which is currently lacking. When given at tolerable doses, the potential for side-effects like ketoacidosis are theoretically possible, so human studies are needed to assess at what dose and time interval exogenous ketones should be administered.

The first ketone esters appeared in the late 1970s. Birkhahn and colleagues synthesized a monoester of glycerol and AcAc (monoacetoacetin) for parenteral nutrition. These studies demonstrated that monoacetoacetin induced hyperketonemia comparable to fasted rats at a high dose of 50 g/kg per day (Birkhahn et al., 1977, 1979; Birkhahn and Border, 1978). In an attempt to increase the caloric density of monoacetoacetin, they synthesized both a monoester and triester of glycerol and BHB. Later, Desrochers and colleagues synthesized monoesters and diesters of AcAc with BD, and these had distinctly different pharmacokinetic profiles – i.e., they elevated both AcAc and BHB (Desrochers et al., 1992, 1995). Pigs given oral boluses of the ketone ester R,S-1,3-butanediol acetoacetate diester (BD-AcAc2), at 15% of the daily caloric requirement exhibited a peak total ketone level of 5 mM within 30 min, before slowly returning to baseline after several hours (Desrochers et al., 1995). No deleterious side-effects were observed through intragastric or high-dose IV administration, including an absence of pathological hypoglycemia and acidosis. These and other ketone esters have demonstrated an ability to induce a dose-dependent hyperketonemia (1–7 mM) in mice, rats, dogs, and humans (Figure 1; Ciraolo and Previs, 1995; Sylvain et al., 1995; Brunengraber, 1997; Puchowicz et al., 2000; Srivastava et al., 2012). In a 28-day study, a daily intragastric gavage (5 gm/kg body weight) of BD-AcAc2 induced significantly elevated blood ketone levels and significantly reduced blood glucose levels without significantly altering blood triglyceride or lipoprotein levels (Kesl et al., 2015). In a 15-week chronic feeding study, the BD-AcAc2 was administered to Sprague Dawley rats in low-dose (10 gm/kg/day) (LKE) and high-dose (25 g/kg/day) (HKE) *ad libitum* protocols. Serum clinical chemistry of both LKE and HKE did not reveal any alterations in markers of kidney and liver function compared to rats fed standard chow (Poff et al., 2016), suggesting that chronic high-dose feeding was without overt toxicity. Similarly, Clarke et al. (2012) demonstrated the safety of a BD-backed BHB monoester in rats and humans, and this has also been documented in a recent case study of Alzheimer’s disease, where the subject consumed a relatively high dose (20–30 g) thrice daily over 20 months (Srivastava et al., 2012; Newport et al., 2015).

The anti-seizure effects of ketone esters were first reported in a unique seizure model which uses hyperbaric hyperoxia (HBO) to reliably induce epileptic-like (i.e., tonic-clonic) seizures in normal rats, a condition known as CNS oxygen toxicity (CNS-OT). The rats in this study were eating standard rodent chow with abundant carbohydrate (>60%) before induced into hyperketonemia. A single oral dose of the ketone ester BD-AcAc2 induced rapid (within 30 min) and sustained (>4 h) ketosis (>3 mM BHB and >3 mM AcAc, 0.5 mM ACE) and prolonged the latency to seizures by 574% (Figure 1) (D’Agostino et al., 2013). Elevations in AcAc and ACE levels were necessary for producing the anti-seizure effects in this particular model of tonic-clonic seizures. BD alone (not in ester form) elevated blood BHB levels (>5 mM) but did not significantly alter AcAc or ACE levels, nor did it prolong the latency to seizure induction. This encouraging response prompted preliminary investigations into preventing or delaying seizures with ketogenic supplements in a variety of transgenic rodent and chemical-induced seizure models.

Pentylenetetrazol (PTZ) is a GABA_A_ receptor antagonist and epileptogenic agent that is used to induce seizures in rodents for preclinical development of anti-seizure therapies. In a study by Coppola and colleagues, the dosage threshold for seizure induction by PTZ was assessed in control (water) and KE-treated rats (Viggiano et al., 2015). A single oral dose (4 gm/kg body weight) of BD-AcAc2 elevated blood BHB to 2.7 mM and increased the threshold of PTZ-evoked seizures from 122 ± 6 mg/kg to 140 ± 11 mg/kg. Although AcAc was not measured in this study, the KE was BD-AcAc2 which produces an approximate 1:1 ratio of BHB and AcAc in the blood. Thus, it can be assumed based on PK data (D’Agostino et al., 2013) that the concentration of AcAc was >2 mM.

More recently, the anti-seizure effect of ketone ester treatment has been evaluated in other Sz models. For example, it has been demonstrated that intragastric administration (gavage) of ketone supplements, such as KE, decreased the absence epileptic activity (spike-wave discharges: SWDs) in WAG/Rij rats (Kovacs et al., 2017). The increase in BHB may exert its therapeutic effects on neurological diseases via modulation of inflammatory systems (Newman and Verdin, 2014; Youm et al., 2015; Yamanashi et al., 2017), which are implicated in the pathophysiology of absence epilepsy (Kovacs et al., 2006, 2011; Tolmacheva et al., 2012; Russo et al., 2014). For example, BHB decreased the expression of NLRP3, ASC, caspase-1 and IL-1β (Bae et al., 2016), attenuated release of IL-1β in human monocytes (Youm et al., 2015) and mitigated stress-induced increase in TNF-α and IL-1β in the hippocampus (Yamanashi et al., 2017). Moreover, BHB attenuates the LPS-evoked increase in IL-1β and TNF-α level, as well as LPS-generated increase in COX-2, IL-1β, and TNF-α mRNA expression in BV-2 cells, likely via inhibition of NF-κB signaling (Fu et al., 2014). It was also demonstrated that BHB may decrease inflammatory processes (e.g., expression of COX and IL-1β) via its G-protein-coupled receptor 109A (GPR109A), which evoked inhibitory influence on NF-κB signaling pathway in microglial cells (Fu et al., 2015; Graff et al., 2016). Thus, ketosis suppresses inflammatory signaling (e.g., NLRP3/TLR4/IL-1R/NF-κB) signaling pathways and proinflammatory cytokines/enzymes (e.g., IL-1β and COX-2) that are linked pathophysiologically to epilepsy and other seizure disorders. Interestingly, BHB induced suppression of inflammation was independent of TCA cycle oxidation, and is thus independent of its function as an energy metabolite. Furthermore, the anti-inflammatory changes associated with BHB were not dependent on AMPK signaling, reactive oxygen species (ROS), glycolytic inhibition, UCP, or SIRT2 signaling, further validating its function as a signaling metabolite with potential anti-seizure function. Indeed, other research has shown that inhibition of the NLRP3 inflammasome mitigates the severity of numerous inflammatory diseases, including atherosclerosis, type 2 diabetes, Alzheimer’s disease, and gout, among others (Martinon et al., 2006; Duewell et al., 2010; Vandanmagsar et al., 2011; Heneka et al., 2013; Youm et al., 2013, 2015). It is also well established that proinflammatory mediators evoke epileptogenic and ictogenic properties following traumatic brain injury (Webster et al., 2017), and thus ketogenic supplementation like BD-AcAc2 that target these inflammatory pathways hold potential for treating post traumatic epilepsy, especially penetrating brain injuries where neuroinflammation is thought to trigger seizure occurrence.

In addition to classical seizure models, the seizure-prone Ube3a m-/p+ mouse model of Angelman Syndrome was studied by supplementing BD-AcAc2 in the food *ad libitum* for 8 weeks (Ciarlone et al., 2016). The KE therapy improved motor coordination, learning and memory, and synaptic plasticity and in AS mice, as well as suppressed Sz frequency and severity. The kainic acid-induced mouse seizure model was also studied. KE increased latency to Sz, decreased Sz activity, and decreased Sz severity. Interestingly, the KE altered brain amino acid metabolism in AS treated animals by increasing levels of glutamic acid decarboxylase (GAD) 65 and 67 (Ciarlone et al., 2016), thus shifting the neuropharmacology of the brain to favor a higher GABA/glutamate ratio. These pre-clinical findings suggested that KE supplementation produces sustained ketosis and ameliorates many symptoms of AS, including seizure activity. Pre-clinical animal studies with exogenous ketone supplementation therapy have inspired human clinical trials in patients with Angelman syndrome (ClinicalTrials.gov Identifier: NCT03644693) and a wide variety of other neurological and metabolic disorders (e.g., NCT03659825, NCT03531554, NCT03226197, NCT03011203, NCT03889210 NCT03878225).

## Medium Chain Triglycerides

Ketogenic fatty acids such medium-chain triglycerides (MCTs) have been a therapy for intractable childhood epilepsy since the early 1970s (Huttenlocher et al., 1971). MCTs are rapidly absorbed, energy dense (8.3 calories/gram), water-miscible, tasteless, and have a much greater ketogenic potential than long chain fatty acids, Huttenlocher et al. (1971) making them an ideal alternative fat source for the KD. Commercial MCT oil is comprised primarily of caprylic acid (C8:0, octanoic acid) and capric acid (C10:0, decanoic acid), and these are absorbed directly into the bloodstream via the hepatic portal vein without the need for bile or pancreatic enzymes for degradation. MCT-induced ketosis (up to 1 mM βHB) occurs independent of carbohydrate or protein consumption, but is currently limited in clinical usage due to gastrointestinal (GI) side effects associated with the dose needed to produce therapeutic ketosis (approximately 40 g/day) (Huttenlocher, 1976). Similarly, the original MCT-based KD allowed 60% of its energy from MCTs, but the reported GI distress in some children (Huttenlocher, 1976; Trauner, 1985; Sills et al., 1986; Mak et al., 1999) lead to a modified MCT-based (30%) KD that induced lower levels of ketosis (Neal et al., 2009).

Several pre-clinical studies have demonstrated that specific MCTs (e.g., C10:0) may have anti-seizure properties through a mechanism of action independent of ketone metabolism and signaling (Chang et al., 2015; Augustin et al., 2018). Oral administration of 4-ethyloctanoic acid (4-EOA) increased Sz control and Sz threshold in several murine Sz models, including the 6 Hz psychomotor Sz model, the maximal electroshock test (MEST), the s.c. metrazol Sz threshold test, and the corneal kindled mouse model (Chang et al., 2015). Capric acid (C10 MCFA) increased Sz threshold in the 6 Hz psychomotor and MEST Sz models, but did not affect outcome in i.v. PTZ-induced Sz (Wlaz et al., 2015). And caprylic acid (C8 MCFA) increased Sz threshold in the 6 Hz psychomotor and i.v. PTZ-induced Sz models, but not in the MEST model (Wlaz et al., 2012).

The addition of MCTs to ketone esters or ketone salts may offer a novel way to improve or further augment their anti-seizure/neuroprotective potential (Ari et al., 2018). A combination of BHB salts and MCT oil has been administered in ratios of 1:1 to 1:2 mixtures. Formulating in this way allows for rapid and sustained elevation of ketosis by delivering exogenous ketones while simultaneously stimulating endogenous ketogenesis with MCTs. In addition, the combination formulation allows for a lower dosing of the components as compared to administering the individual supplements, thus reducing potential for side effects (gastric hyperosmolality) and resulting in a distinct blood ketone profile that is sustained over a longer period of time (D’Agostino et al., 2015). In a 28-day study in rats, the combination of MCT with a 50% Na^+^/K^+^ βHB salt mixture (in 1:1 solution) significantly elevated and sustained blood ketone levels and reduced blood glucose levels in a dose-dependent manner (Kesl et al., 2016). In a 15-week study, Sprague Dawley rats were administered a 1:1 mixture of Na^+^/Ca^+2^ ketone salt + MCT oil (20% by weight which resulted in approximately ∼25 g/kg/day) in their food fed *ad libitum*. The combination-supplemented rats had significantly sustained and elevated blood ketone levels at weeks 3, 4, 8, 10, and 13 (Kesl et al., 2014). Exogenous ketone supplements have typically been studied as a single stand-alone supplement, but the unique combination MCT added to ketone salts or ketone esters appears to have pharmacokinetic advantages and favorable behavior effects (Ari et al., 2016; Kesl et al., 2016). Formulating specific supplements will likely enhance the tolerability, absorption, peak and sustained levels of ketones in the blood, which may also translate to greater therapeutic potency and anti-seizure efficacy (Kovacs et al., 2017; Ari et al., 2018).

## Ketone Salts

The recent commercialization of ketone salt supplements has fueled interest in these formulations for general health and wellness, but their clinical efficacy for seizure disorders remains largely unknown. Originally, researchers attempted to administer oral BHB or AcAc in their free acid forms; however, this was prohibitively expensive and ineffective. Subsequently, it was suggested to buffer the free acid of BHB with sodium, but it was feared that sodium overload would occur at therapeutic levels of ketosis. Furthermore, the existing data does not support elevating BHB alone will effectively prevent seizures in animal models (Bough and Rho, 2007). A study showed that oral administration D,LBHB (racemic BHB) treatment for multiple acyl-CoA dehydrogenase deficiency (MADD) was remarkably therapeutic for cerebral and cardiac complication in doses from 80 to 900 mg/kg/day (BHB levels 0.19-0.36 mM) in children with the disease (Hove et al., 2003). Similarly, a successful treatment of severe cardiomyopathy in a pediatric patient with glycogen storage disease type III with the KD and racemic ketone (D/L-BHB) sodium salts was achieved (Valayannopoulos et al., 2011). Although these results are compelling, these protocols would require ingesting between 5.6 and 6.3 g sodium/day for a 70 kg man. Considering the potential safety effects of such a large sodium load, the costs of the administration of Na^+^/βHB salts to achieve ketosis made this approach unrealistic (Veech, 2004). Since any physiological electrolyte (Na^+^, K^+^, Ca2^+^, Mg^2+^) readily ionically bonds with BHB, it was determined that a balanced ketone electrolyte formulation would be safer and more feasible for sustaining therapeutic ketosis. Over the last few years chemists have synthesized these balanced ketone electrolyte formulations and numerous studies have been published in animal models (Kephart et al., 2017) and humans (Stubbs et al., 2017, 2018a). A few pre-clinical studies have evaluated the anti-convulsant effects of ketone salts delivered exogenously by i.p. injection (Figure 1). D-BHB i.p. increased latency to Sz in the Frings audiogenic-induced Sz mouse model (Rho et al., 2002). I.p. D/L-BHB decreased Sz activity in suckling rats given flurothyl (Minlebaev and Khazipov, 2011), and chronic, but not acute, i.p. administration of D-BHB decreased Sz frequency in NMDA-induced rat Sz model (Yum et al., 2015). Similarly, s.c. delivery of BHB via osmotic pump decreased Sz frequency *in vivo*, and decreased spontaneous Sz-like events (SLE) in hippocampal slices, from *Kcna1*-null mutant mice (Kim et al., 2015). At the time of this writing, millions of doses of commercially available ketone salt products have been purchased and consumed, and no severe adverse reactions have been reported on the FDA website. Widespread use of these products, and better formulations for palatability and tolerability, may help to advance their clinical acceptance and implementation as a means to induce and sustain therapeutic ketosis. Regardless, significant clinical evaluation of the safety and efficacy of chronic ketone salt consumption for seizure disorders has yet to be published.

## Limitations

There are a number of studies that highlight the limitations to ketone salt and ketone esters that are available commercially or for research applications. These limitations are primarily due to gastrointestinal symptoms associated with aversive taste or osmotic load in the GI tract (Leckey et al., 2017; Fischer et al., 2018). Future studies need to assure that the ketone supplement formulations are well tolerated and provide an ideal pharmacokinetic profile of sustained ketone elevation before such supplements are evaluated in humans (Stubbs et al., 2018b). Of relevance to this review, it is important to highlight that while pre-clinical studies have demonstrated that ketone supplements offer promising anti-convulsant effects in a variety of animal models, very little work to date has been published evaluating their potential anti-seizure efficacy or utility in humans.

Historically, issues of palatability and tolerability have limited the clinical investigation of exogenous ketone supplements. More recently, commercialization of ketone salt and MCT oil products have resulted in formulations that are pleasant to the taste and unlikely to elicit significant gastrointestinal distress unless overconsumed. For more potent formulations, such as ketone esters, it has proven more difficult to mask these flavoring issues and GI effects. In fact, in a recent study evaluating the potential utility of BD-AcAc2 as an ergogenic agent in cyclists, performance was impaired in the group receiving the ketone ester (Leckey et al., 2017). However, all of the study participants experienced notable gastrointestinal discomfort from consuming the supplement, confounding interpretation of the results (Stubbs et al., 2018b). Still, some ketone ester formulations have overcome these major obstacles, resulting in commercially viable products with a much improved taste and GI effect profile, such as the beta-hydroxybutyrate monoester (Stubbs et al., 2018a). Ongoing efforts to optimize other ketone esters such as BD-AcAc2 is promising, and likely to result in a similar commercialized product soon. Combining multiple ketogenic agents in a controlled-release formulation appears to be a promising direction (Meidenbauer et al., 2015).

Another major issue that will need to be addressed to move ketogenic agents into the clinic is establishing an understanding of their appropriate method of administration and dosing regimen. As described, different formulations of ketone supplements elicit markedly different pharmacokinetic profiles, with variable concentrations and durations of blood BHB and/or AcAc produced. Currently, a single dose of most commercially available ketone salt formulations can elevate blood BHB by approximately 1 mM for 1–3 h (O’Malley et al., 2017). If sustained ketosis is required for therapeutic effects, numerous daily doses of these agents would be needed, which may prove to be logistically difficult. Furthermore, as ketone salt formulations often contain large quantities of electrolytes, namely sodium, frequent dosing may present challenges in complying with the recommended maximum daily intake guidelines for these minerals. MCT oil alone can elevate blood ketones modestly (∼0.5 mM) (Courchesne-Loyer et al., 2015), but can also produce significant GI distress at large or frequent doses, especially in naïve patients. Pre-clinical work in rats suggest that adding MCTs to a ketone salt formulation may provide a method to improve the sustained elevation of ketosis with less side effects (Kesl et al., 2016), and therefore may provide a viable option for clinical use. Ketone esters appear to be able to elevate blood ketones to higher concentrations and for longer periods of time than any other currently available ketone formulation (Stubbs et al., 2017), but carry with them a greater need for safety testing and a higher risk of inducing hyperketonemia if overconsumed. In addition to these obstacles, the background diet of the patient would need to be considered, as it may affect the clinical profile of exogenous ketone therapy. If an individual is consuming a KD, exogenous ketone supplements may increase levels of ketosis overall, but also could potentially reduce rates of endogenous fatty acid oxidation and ketogenesis which may play a role in the diet’s therapeutic efficacy. Thus, these and other details regarding dosing protocols will need to be established for individual clinical applications. We expect that optimal protocols will depend on the type of ketone formulation being utilized and the specific condition being treated, similar to the use of any pharmaceutical agent. Efforts to optimize the composition and delivery of exogenous ketone supplements are ongoing – such as efforts to improve palatability, reduce GI side effects, and prolong sustained ketosis – and will likely improve tolerability and utility of these agents with time.

## Conclusion

Considering the multifaceted therapeutic effects and success of the KD for seizure disorders, the goal of many ketone supplement researchers has often been described as creating “the KD in a pill.” As such, exogenous ketone supplements are being developed as an alternative or adjuvant method of inducing therapeutic ketosis without the need for a strict dietary regimen. Considering the promising results of the recent pre-clinical studies described here, along with advancements in optimizing ketone supplement formulations, it is possible that many of the seizure conditions which are known to benefit from the KD could receive some benefit from exogenous ketone supplementation by elevating blood ketones and lowering blood glucose. Importantly, if ketone supplements prove safe and efficacious in human trials, they may provide a tool for achieving ketosis in patients who are unable, unwilling, or uninterested in consuming a classic KD, modified Atkins diet, or LGIT. Ketone supplementation may also help circumvent some of the difficulties associated with dietary therapy, as it allows for a rapid dose-dependent induction of ketosis, which can be sustained with prolonged consumption and monitored precisely with commercially available technologies (e.g., blood ketone meters). Simultaneously, it could provide patients with the opportunity to reap the benefits of therapeutic ketosis without the practical and social difficulties of a highly restrictive diet. Moreover, these agents may represent a means to further enhance or optimize existing ketogenic therapies by supplying a form of non-glycemic calories that improves parameters (e.g., GKI) that are associated with therapeutic benefits.

Research on the potential applications of ketone supplementation is rapidly growing, and there are currently several registered clinical trials evaluating their safety and efficacy in a variety of conditions, including healthy adults, athletes, and patients with various diseases including Alzheimer’s, Parkinson’s, Type 2 Diabetes Mellitus, and more^1^. Encouragingly, clinical studies evaluating these agents in seizure disorders are beginning to emerge. An ongoing trial in Angelman syndrome – a genetic neurodevelopmental disorder characterized intellectual and developmental disability and seizures – is evaluating the use of a fat-based nutritional formulation containing exogenous ketones to support nutritional needs of this patient population (NCT03644693). As a secondary outcome measure, the investigators will also be tracking changes in EEG and seizure activity. Anecdotal reports of individuals consuming commercially available ketone supplements have suggested that some individuals experience a subjective improvement in seizure activity with their use, despite the fact that some of the more potent formulations, such as BD-AcAc2, are not yet commercially available. Regardless, it is important to highlight that there is a lack of published clinical work demonstrating efficacy of such agents in patients with seizure disorders, and the relationship between blood ketone elevation and the protective effects of ketosis on seizures is unclear. Thus, further research is needed to fully investigate the molecular mechanisms, clinical utility, and feasibility of exogenous ketone supplements as a method of inducing therapeutic ketosis for managing seizures.

## Disclosure

We confirm that we have read the journal’s position on issues involved in ethical publication and affirm that this report is consistent with those guidelines. JR has served as a paid consultant for Accera Pharma, Xenon Pharmaceuticals, Danone Nutricia, and Ajinomoto United States. DD’A and AP receive travel reimbursement and honoraria for speaking at scientific and clinical conferences. AP has served as a paid consultant for Pruvit Ventures, LLC.

## Author Contributions

AP, JR, and DD’A contributed to the literature review, figure and table design, and writing of the manuscript. All authors read and approved the submitted version.

## Conflict of Interest

International Patent # PCT/US2014/031237, University of South Florida, DD’A, S. Kesl, P. Arnold, “Compositions and Methods for Producing Elevated and Sustained Ketosis.” USF Ref. No: 16B128 (provisional patent); C. Ari, DD’A, J. B. Dean. Technology Title: “Delaying latency to seizure by combinations of ketone supplements.” DD’A is co-owner of the company Ketone Technologies, LLC, providing scientific consulting and public speaking engagements about ketogenic therapies. The company obtained an option agreement from the University of South Florida on the non-provisional patent No. 62/310,302 “Methods of increasing latency of anesthetic induction using ketone supplementation.” These interests have been reviewed and managed by the University in accordance with its Institutional and Individual Conflict of Interest policies. AP is a co-founder and owner of Metabolic Health Initiative, LLC, a company that provides educational content and seminars related to ketogenic and metabolic therapies. AP is also a co-owner of Poff Medical Consulting & Communications, LLC, a scientific consulting company. JR is a co-founder and shareholder of Path Therapeutics, Inc., based on Calgary, AB, Canada.

## Footnotes

1 www.clinicaltrials.gov
